# Distalization of mandibular molar with iatrogenic root fracture in Class III malocclusion: a case report

**DOI:** 10.1186/s12903-024-05174-w

**Published:** 2024-11-15

**Authors:** Hyojin Kim, Hyeon Gi Hong, Ji Yoon Jeon, Kee-Joon Lee

**Affiliations:** Department of Orthodontics, Institute of Craniofacial Deformity, College of Dentistry, Yonsei University, 50-1 Yonsei-ro, Seodaemun-gu, Seoul, 03722 Korea

**Keywords:** Tooth injuries, Orthodontic mini-implant, Mandibular arch distalization

## Abstract

**Background:**

Placement of interradicular orthodontic miniscrews poses a potential risk of root damage, including superficial root contact and root fracture. This case report describes the iatrogenic root-injured tooth movement of a 27-year-old male with skeletal Class III malocclusion as nonsurgical orthodontic treatment.

**Case presentation:**

An orthodontic miniscrew between the mandibular right first and second molars perforated the distal root of the mandibular first molar. A root fracture was discovered 4 months after miniscrew placement. Owing to the potential risk of ankylosis related to surgical intervention, a direct orthodontic distalizing force was applied towards the fractured distal root segment without additional treatment, resulting in considerable movement of the fractured tooth with maintaining tooth vitality. However, gradual root resorption of a fractured tooth with a separate root segment was observed. The mandibular arch distalization of skeletal Class III malocclusion was successfully performed and retained for 3 years 8 months with stable occlusion.

**Conclusions:**

This case reveals a clinical remedy when root movement of a tooth with root fracture is indicated. The use of extra-alveolar miniscrews or miniplates can be considered for mandibular arch distalization to prevent potential root injuries caused by miniscrew placement.

**Clinical trial number:**

Not applicable.

## Background

Orthodontic miniscrews have been effectively and widely used to extend the limits of tooth movement with minimal patient’s cooperation [1]. They have simple placement and removal surgical procedures, multiple placement sites, and a high success rate [1]. As reliable skeletal anchorage devices, orthodontic miniscrews have expanded the scope of nonsurgical orthodontic treatment for adult patients [2].

Despite various clinical applications of miniscrews, complications can occur during miniscrew insertion, tooth movement, and miniscrew removal [1]. Previous studies demonstrated that the proximity of roots to miniscrews is associated with damage to adjacent roots and an increased risk of failure [3, 4]. For safe insertion of miniscrews in the interradicular area, a minimum distance of 1.5 mm is required between the roots in both the maxilla and the mandible [5]. The damage to the adjacent root usually heals spontaneously and does not require further treatment [1, 6]. If the damage is moderate to severe, endodontic treatment or surgical intervention may be necessary [7–9].

A few studies have reported on root fractures caused by miniscrew placement. Recent studies [10, 11] demonstrated that iatrogenic root perforations involving the pulp can be repaired spontaneously, maintaining tooth vitality. However, the outcome of orthodontic treatment including the damaged tooth was not discussed. Therefore, this report described a case of remarkable movement of the damaged tooth after iatrogenic distal root fracture of the mandibular first molar by an orthodontic miniscrew for nonsurgical treatment of a skeletal Class III malocclusion.

## Case presentation

### Diagnosis and etiology

A 27-year-old male patient presented with the chief complaint of relapsed posterior crossbite and sought orthodontic treatment. The patient had undergone orthodontic treatment nine years ago, during which four premolars and third molars were extracted to correct Class III malocclusion. No systemic diseases were observed.

**Fig. 1:**
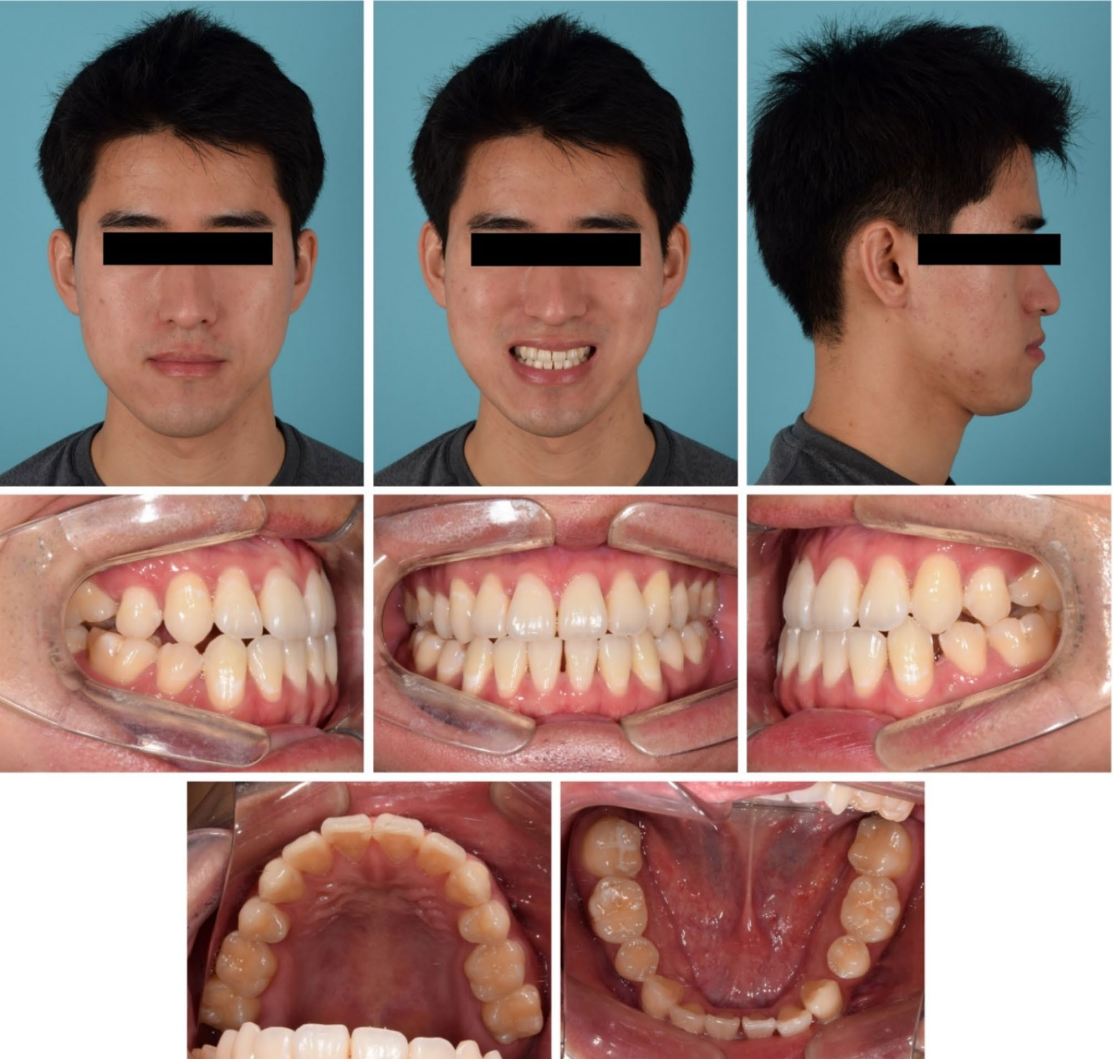
Pre-treatment photographs

**Fig. 2:**
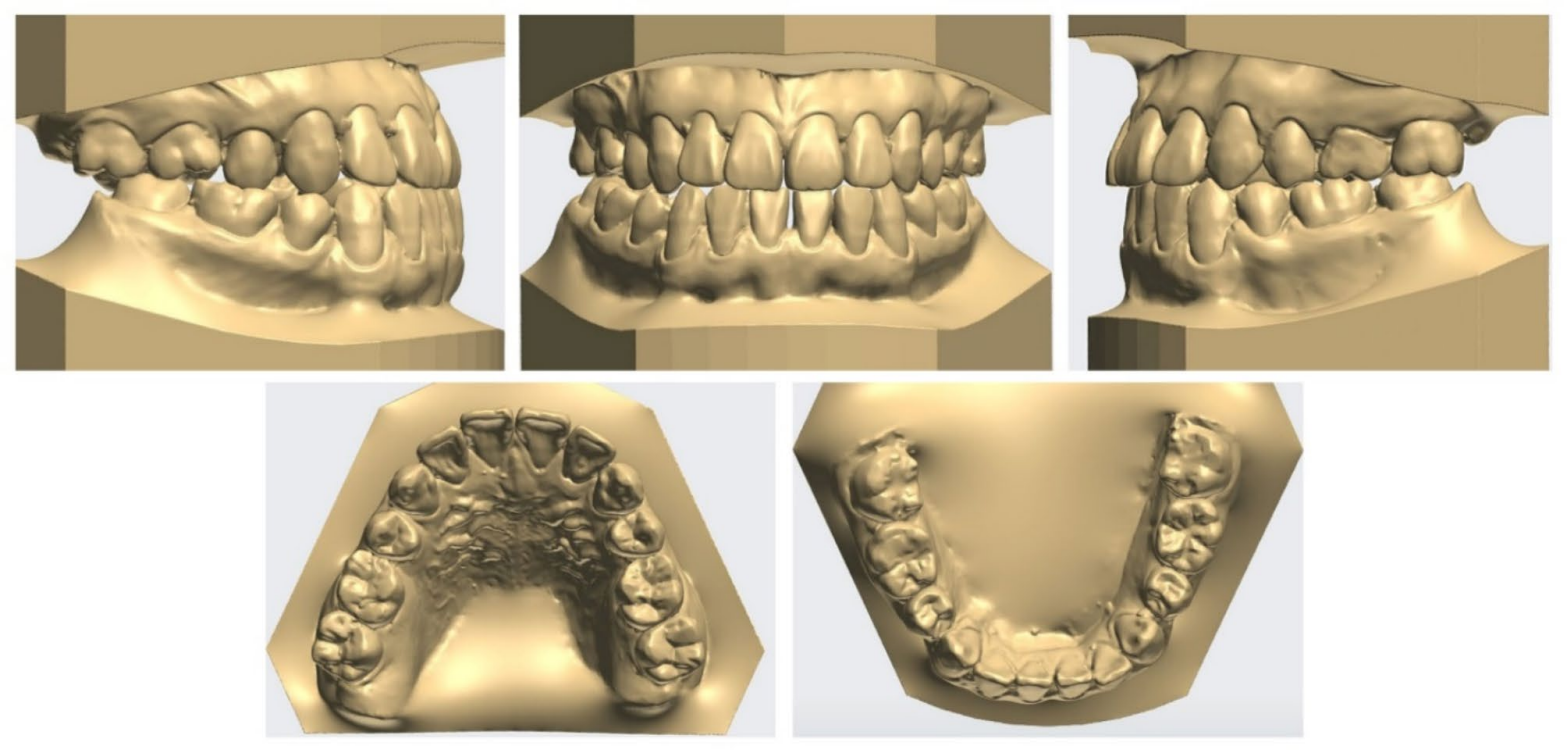
Pre-treatment dental models

Extraoral examination revealed upper lip retrusion and lower lip protrusion. Intraoral examination indicated space relapse in maxillary and mandibular central incisors and mandibular left premolar area. The overjet and overbite were 1.0 mm both. A bilateral posterior crossbite and Class III molar relationship were observed with a narrow maxillary arch. Maxillary dental midline was 1 mm deviated to the right side (Figs. 1 and 2).

**Fig. 3:**
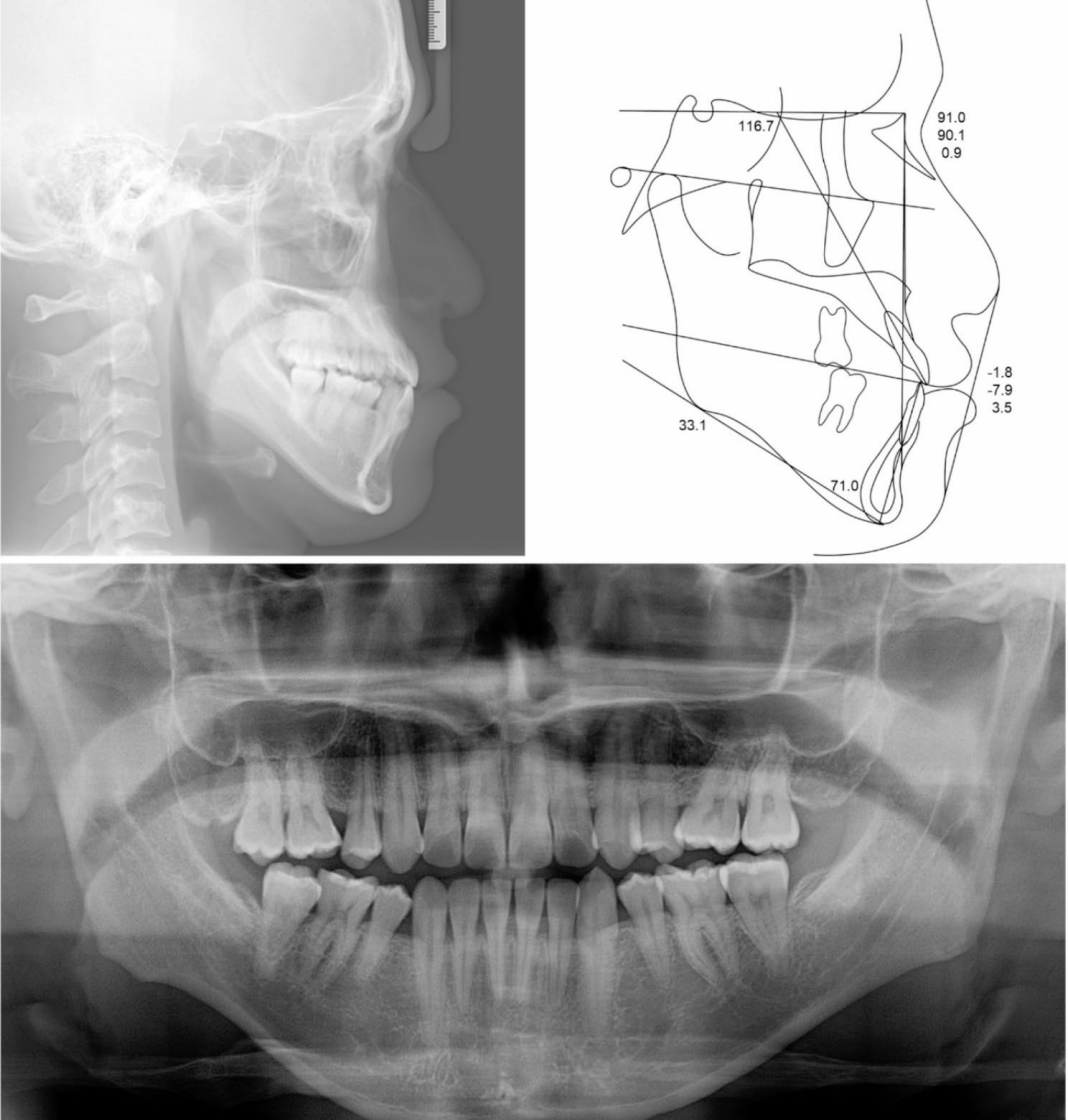
Pre-treatment radiographs and cephalometric tracing

Lateral cephalometric analysis indicated skeletal Class III malocclusion with normodivergent facial profile. Proclined maxillary incisors and reclined mandibular incisors were observed (Table 1; Fig. 3). Panoramic radiograph showed that the four premolars and third molars had been extracted because of a previous orthodontic treatment. In addition, mesial tipping was observed in the maxillary first molars and mandibular right molars (Fig. 3).

**Table 1:** Cephalometric analysis measurements

Measurements	Pretreatment	Posttreatment
SNA (^0^)	91.0	91.9
SNB (^0^)	90.1	90.7
ANB (^0^)	0.9	1.2
Wits (mm)	-7.9	-3.2
SN - GoMe (^0^)	33.1	31.8
Bjork sum (^0^)	393.1	391.8
U1 to SN (^0^)	116.7	125.0
IMPA (^0^)	71.0	74.3
Upper lip to E-line (mm)	-1.8	-3.0
Lower lip to E-line (mm)	3.5	0.2

### Treatment alternatives

Because the patient had previously undergone camouflage orthodontic treatment, orthognathic surgery was suggested to resolve the chief complaint. However, the patient was only interested in nonsurgical orthodontic treatment. Following a cone-beam computed tomography (CBCT) scan to confirm the available space, mandibular arch distalization using orthodontic miniscrews was therefore chosen to achieve a stable occlusion and an esthetic facial profile. Miniscrew-assisted rapid palatal expander was planned to improve the transverse discrepancy.

### Treatment progress

**Fig. 4:**
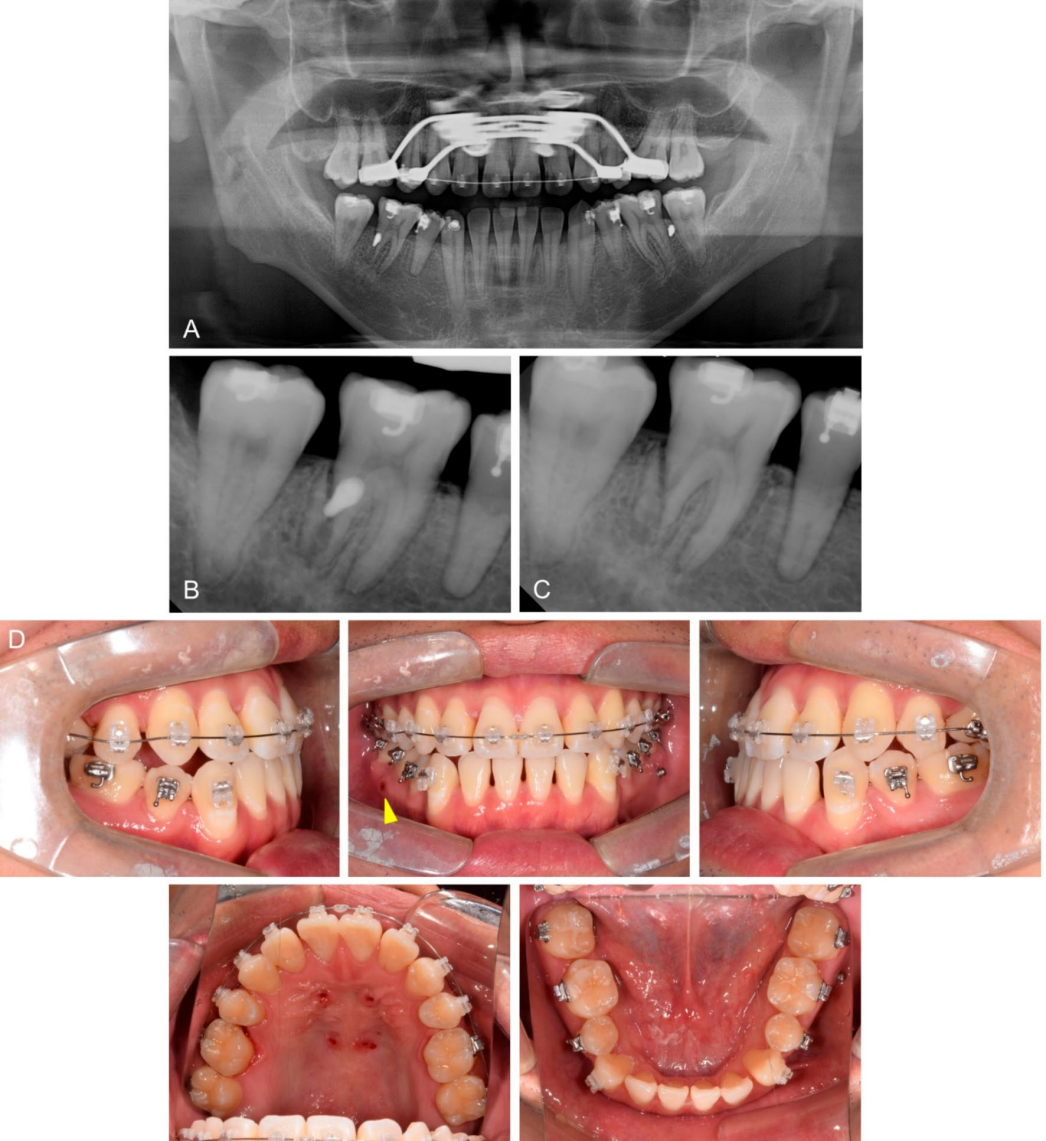
Distal root fracture of the mandibular right first molar by an orthodontic miniscrew. Radiographs and photographs before miniscrew removal (**A**, **B**), and after miniscrew removal (**C**, **D**)

Self-ligating 0.018-inch Roth prescription brackets (Clippy-C; Tomy, Tokyo, Japan) were bonded to the mandibular arch, except for the mandibular incisors. A 0.016-inch nickel-titanium archwire was used as the initial wire for alignment. Retromolar miniscrews were inserted for distalizing the posterior mandibular arch. However, these resulted in soft tissue impingement on both sides and removed. Consequently, orthodontic miniscrews (diameter 1.8 mm x length 7.0 mm; Orlus, Ortholution, Seoul, Korea) were inserted bilaterally in the buccal interradicular space between the first and second molars in the mandible. In the event of interference with the miniscrews, a repositioning of the miniscrews during the orthodontic treatment was considered. A 0.016 × 0.022-inch stainless steel archwire was used as the working wire in the continuous wire sequence of the posterior mandibular arch. A miniscrew-assisted rapid palatal expander was used for dentoalveolar expansion without separating the midpalatal suture as a result. After the expander removal, the same bracket system was bonded for maxillary arch alignment (Fig. 4).

While distal movement of the posterior teeth in the mandibular arch was confirmed on the left side, there was no sign of movement on the right side. A periapical radiograph was taken 4 months after miniscrew insertion to confirm the interference of the miniscrew. The radiograph indicated that the miniscrew had perforated the distal root of the mandibular right first molar with an oblique fracture line (Fig. 4B). However, the patient did not complain of discomfort or pain in the damaged tooth after miniscrew placement. The miniscrew and distalizing orthodontic force were removed immediately on the right side (Fig. 4C). The tooth vitality of the damaged tooth was confirmed by a cold test. An endodontist evaluated the absence of signs of infection and recommended tooth monitoring after 3 months without endodontic treatment.

### Clinical options for the mandibular first molar with root fracture

The fracture of the distal root of the mandibular right first molar was caused by an orthodontic miniscrew during leveling and alignment. Consequently, a decision was made as to whether and how the tooth should be preserved.

#### Hemisection of the fractured distal root

The remaining mesial root of a mandibular first molar has a longitudinal groove that contraindicates the use of a post for dental restoration [12]. Additionally, surgical intervention can lead to tooth ankylosis which limits the distalization of the tooth [13].

#### Removal of the fractured root fragment

Tooth ankylosis can occur during the healing process [13].

#### Extraction

A dental implant is necessary to replace an extracted molar after orthodontic treatment. However, tooth extraction is considered the final option when tooth preservation fails.

#### Monitoring without interventions

The patient did not complain of discomfort or pain before the discovery of the root fracture. No sign of infection was observed after miniscrew placement. Considering presence of the tooth vitality, the damaged tooth was monitored.

**Fig. 5:**
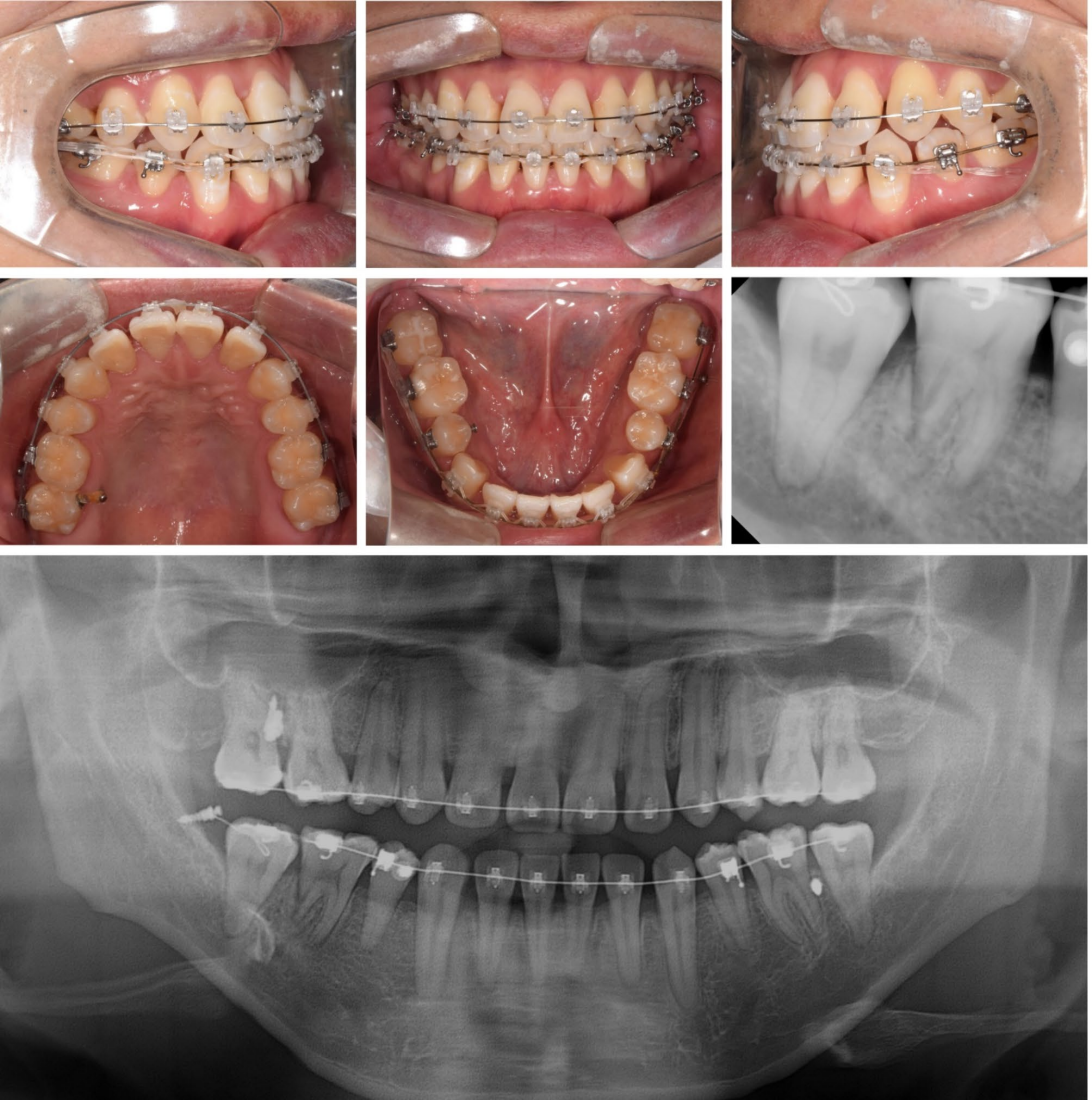
Mid-treatment photographs and radiographs during the mandibular arch distalization without observation period (1 year after the root fracture)

**Fig. 6:**
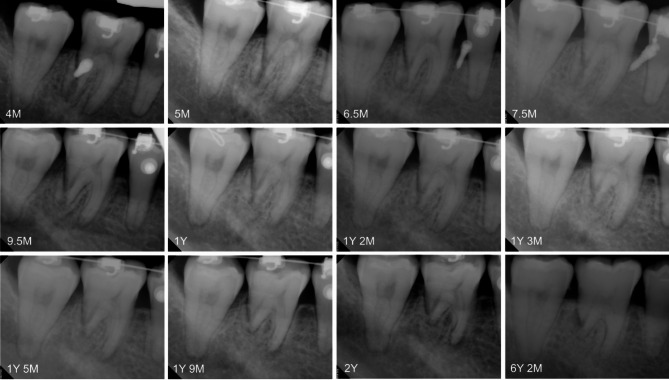
Serial periapical radiographs of the root-injured tooth from 4 months to 6 years 2 months after injury. Gradual root resorption of the damaged tooth with root segment was observed. Years (Y); and Months (M)

The decision was made to preserve the damaged tooth as much as possible. Mandibular molar distalization including the damaged tooth was started immediately with a new miniscrew (diameter 1.8 mm x length 7.0 mm; Orlus, Ortholution, Seoul, Korea) re-insertion on the mandibular right buccal shelf area. The maxillary right second molar was intruded using a palatal miniscrew to correct the posterior occlusion (Fig. 5). The lingual miniscrew between the mandibular right second premolar and first molar was placed for the mandibular right canine control (Fig. 6). The patient complained of intermittent cold hypersensitivity from 10 months after the root fracture. This symptom gradually disappeared in the middle of the tooth movement. Endodontic treatment could be considered at any point during orthodontic treatment if the subjective symptoms of the patient worsen. A series of periapical radiographs were planned to be taken at every two visits to monitor the damaged tooth (Fig. 6).

**Fig. 7:**
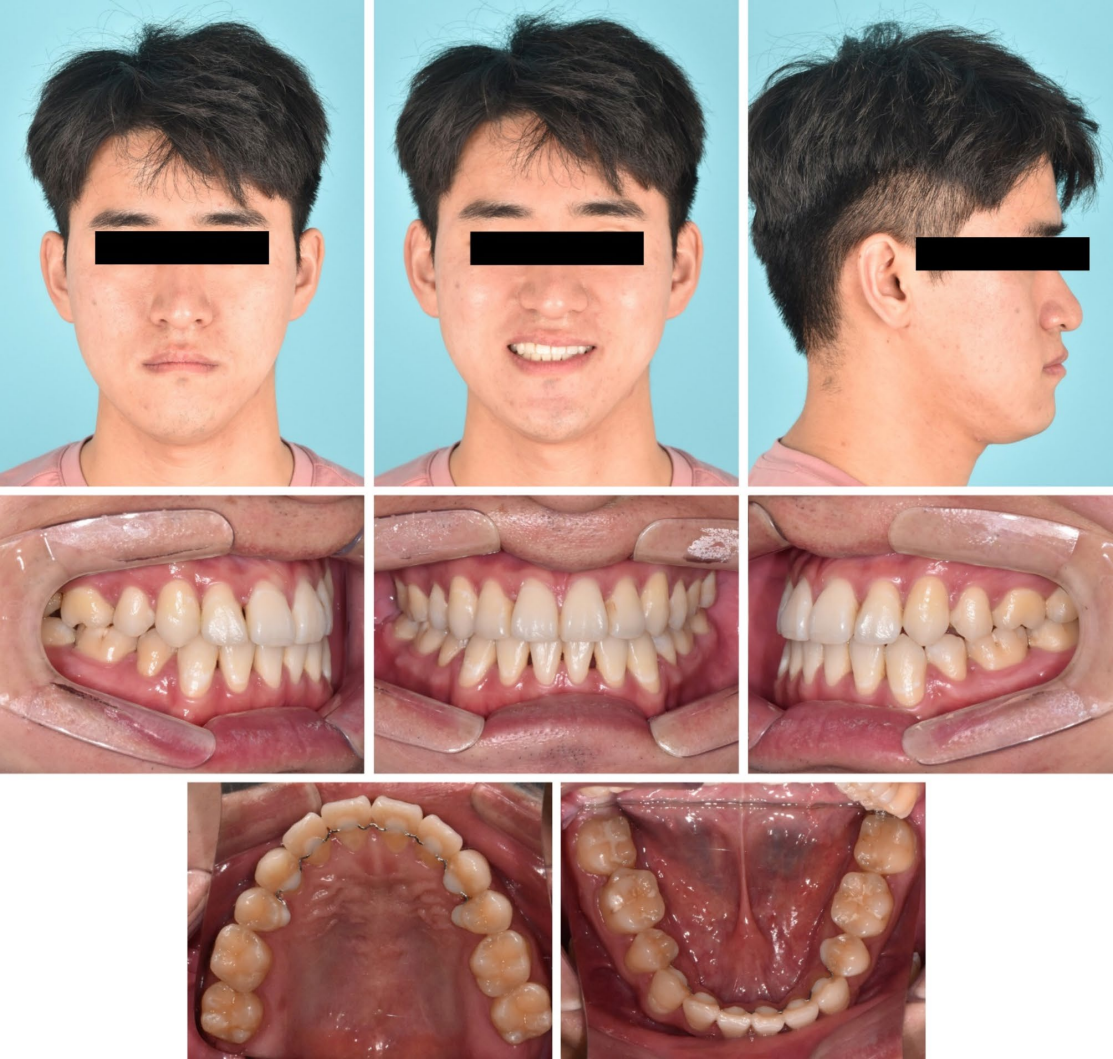
Post-treatment photographs

After 36 months of active treatment, mandibular arch distalization was completed without additional treatment of the fractured tooth. All appliances and miniscrews were removed. Fixed lingual retainers and circumferential retainers were delivered to both arches for retention (Fig. 7).

### Treatment results

**Fig. 8:**
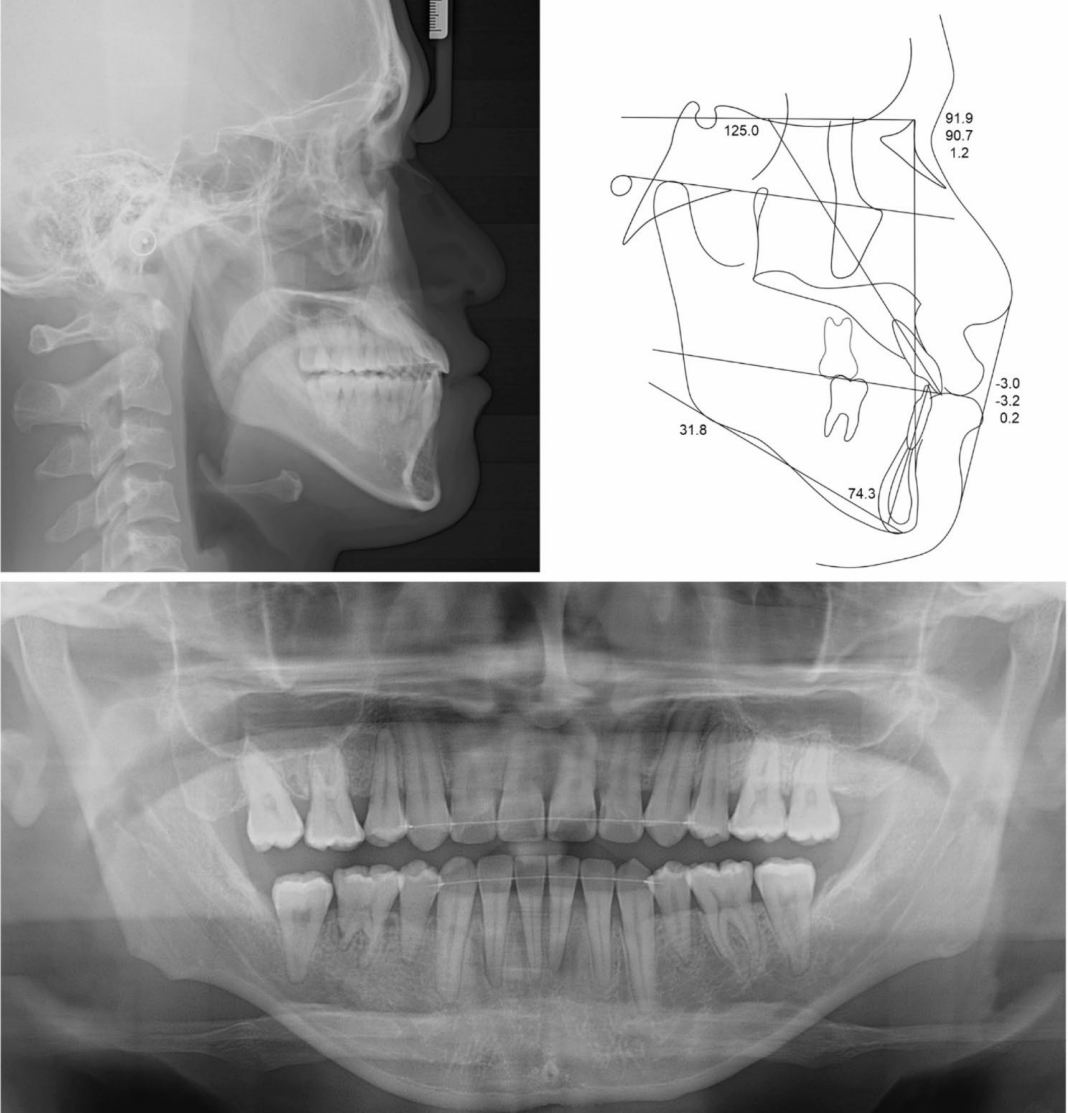
Post-treatment radiographs and cephalometric tracing

After the completion of nonsurgical orthodontic treatment, the patient’s chief complaint related to the posterior crossbite was resolved, and the facial profile improved significantly. As shown in Fig. 7, stable occlusion with adequate overjet and overbite was established through mandibular arch distalization, which included a root-injured tooth. Minor root resorption of the incisors and premolars in both arches was observed (Fig. 8).

**Fig. 9:**
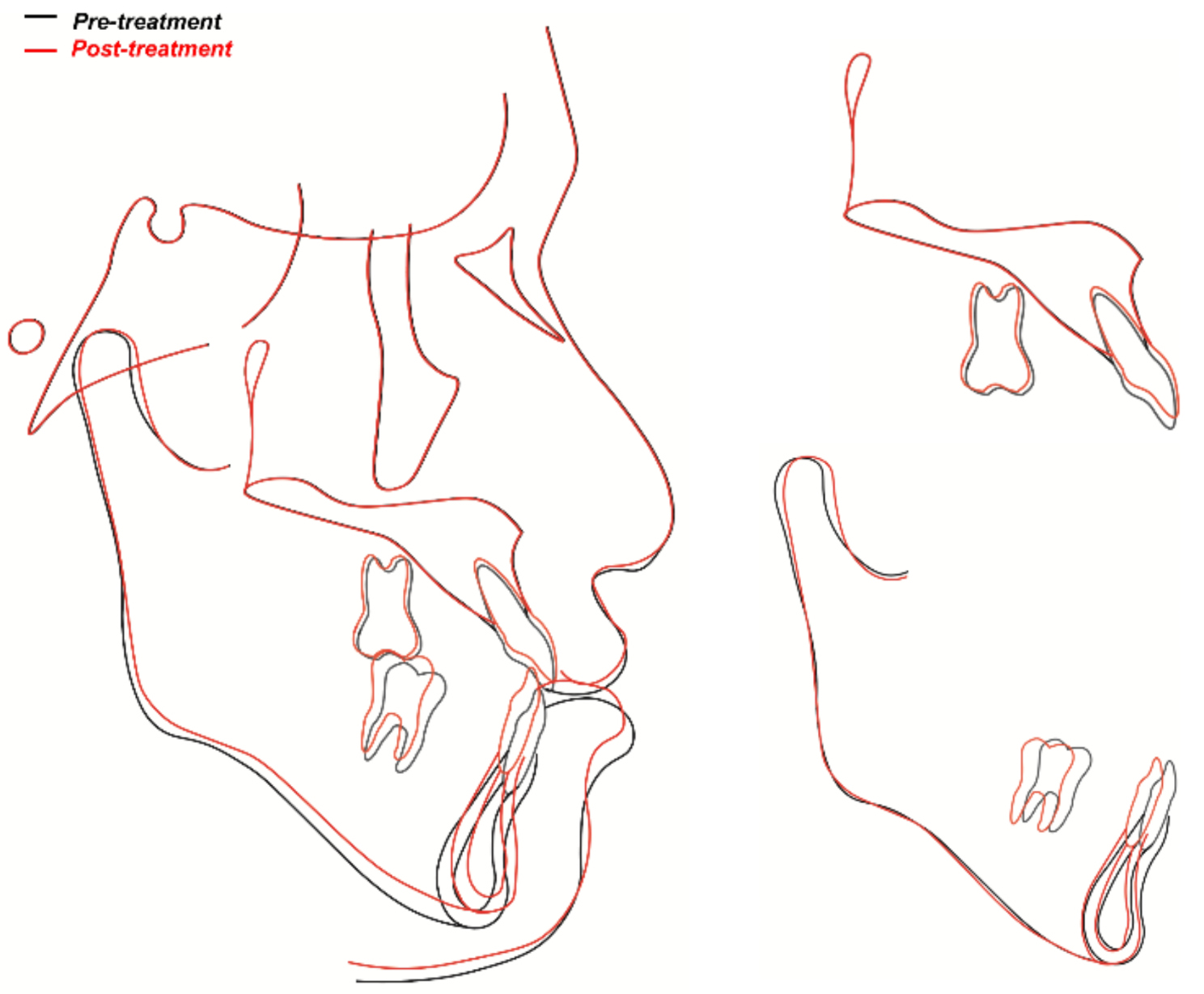
Superimposition images of the pre- and post-treatment cephalometric tracings

**Fig. 10:**
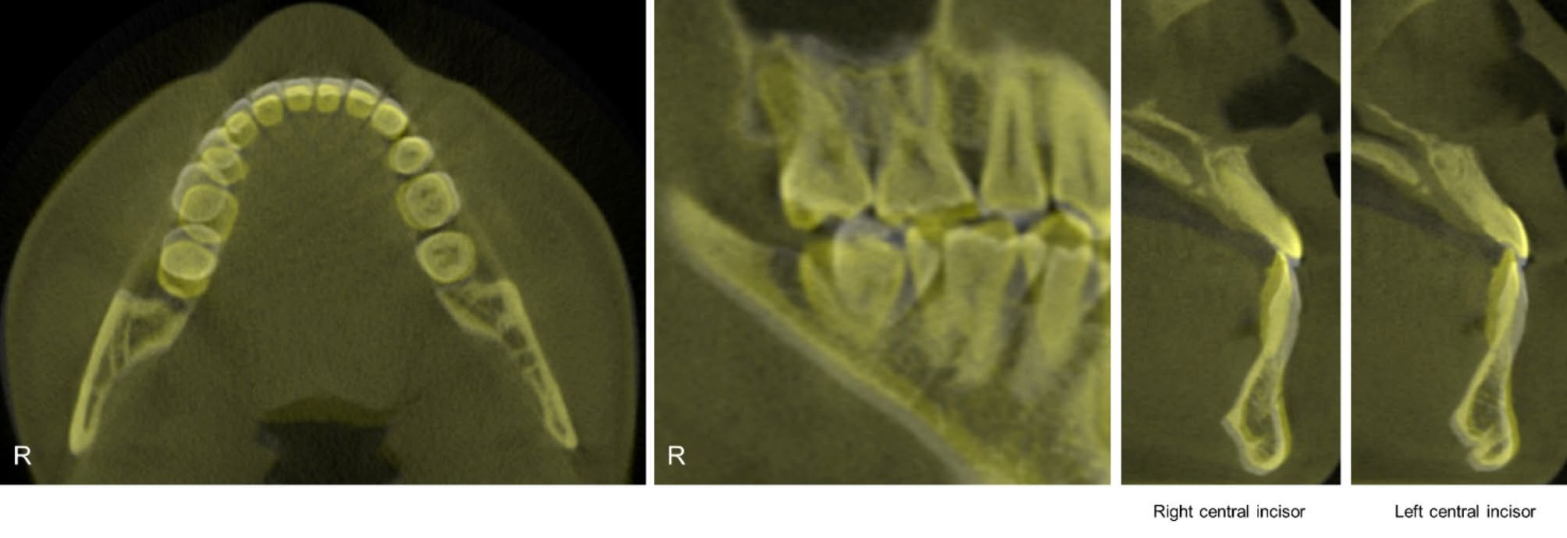
Superimpositions of CBCT images. Distal displacement with mild inclination of the mandibular right first molar was observed. Pre-treatment (White); and Post-treatment (Yellow)

**Fig. 11:**
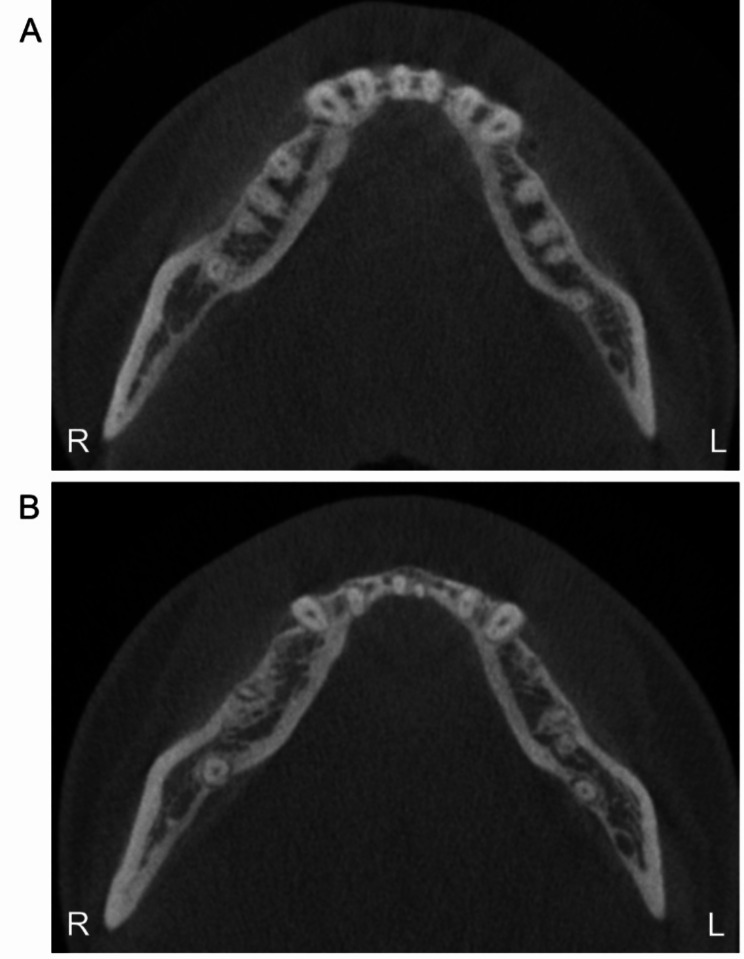
Comparison of CBCT images (Axial). (**A**) Pre-treatment; (**B**) Post-treatment

Post-treatment cephalometric analysis and superimposition revealed a successful distal movement of the mandibular arch with a counterclockwise rotation of the mandible and the occlusal plane (Fig. 9). The mandibular incisors flared slightly (Table 1). Labiolingual proclined maxillary incisors and retracted mandibular incisors improved the facial profile, especially the lower lip. In the post-treatment CBCT images and superimposition, the distal displacement with mild inclination of the fractured tooth was confirmed to be 4 mm at the crown level. The extent of mandibular molar distalization was more prominent on the right side than on the left side (Fig. 10). Additionally, mandibular molar distalization accompanied minor lingual cortex remodeling (Fig. 11).

**Fig. 12:**
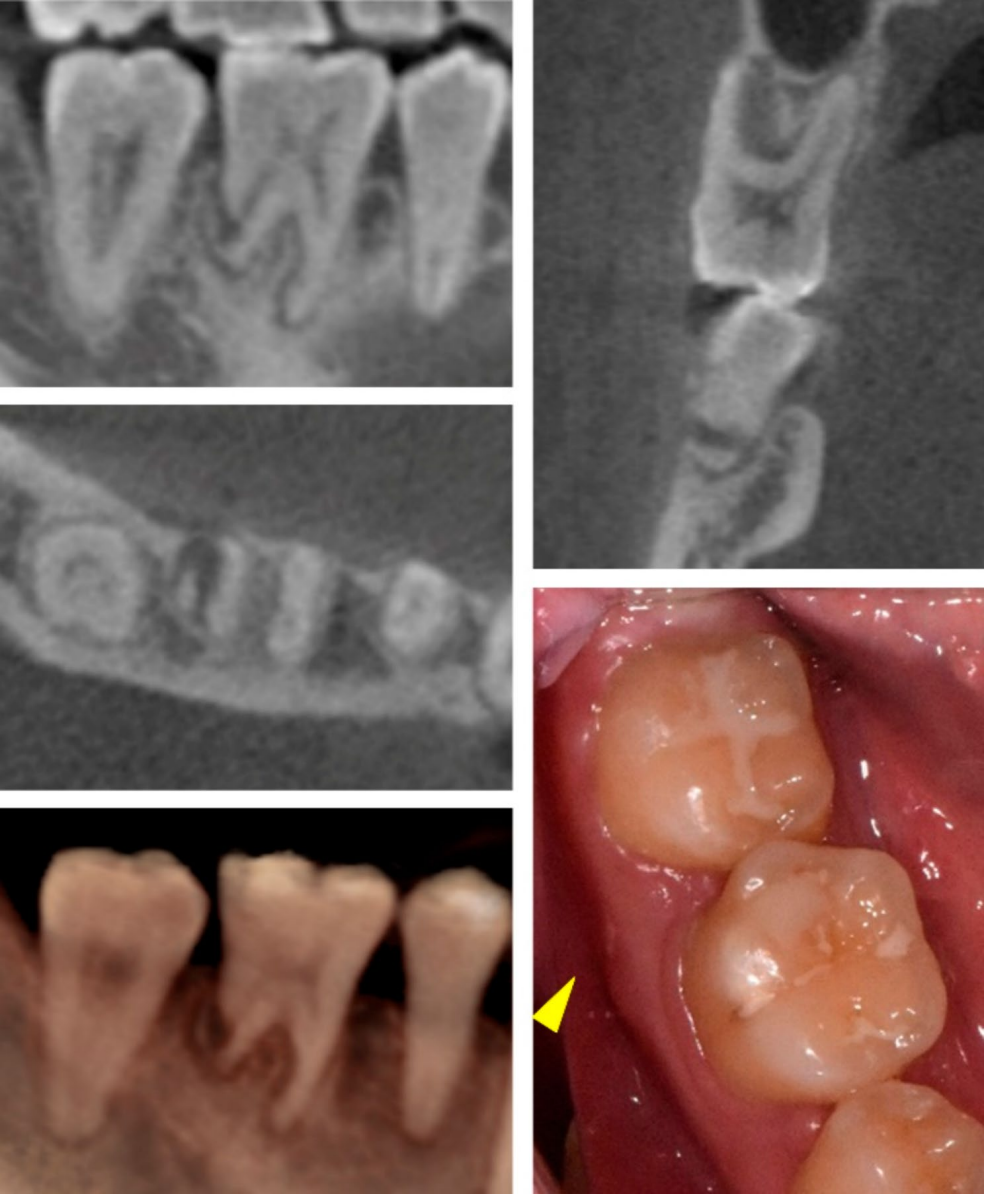
Post-treatment CBCT images and intraoral photograph of the root-injured tooth

Regarding the root fracture, the fragment was separated from the distal root of the mandibular right first molar. In addition to distal root with root fragment, the mesial root was gradually resorbed without periapical lesion during orthodontic treatment (Fig. 6). No evidence of ankylosis or pulp necrosis was observed. Despite the root fracture with pulp involvement, the damaged tooth vitality was maintained at the end of the orthodontic treatment. No bone-like tissue formation was observed between the segment and the tooth body (Fig. 12).

**Fig. 13:**
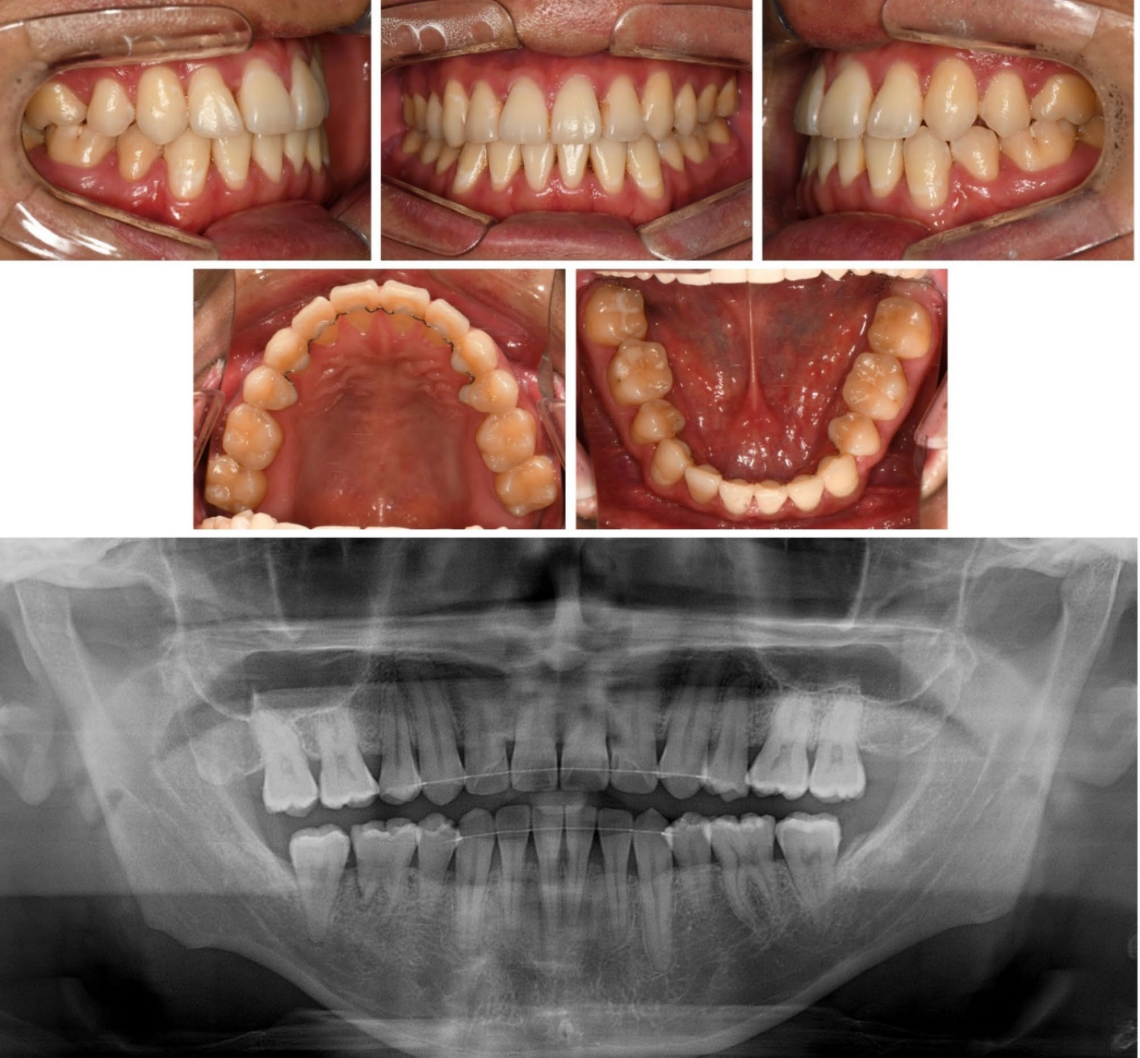
6 years 2 months after the root fracture (3 years 8 months of follow-up) photographs and radiograph

At 6 years 2 months after the root fracture (3 years 8 months of follow-up), the root-injured tooth maintained its vitality with a positive cold test. Normal periodontal ligament (PDL) space and minor pulp obliteration were observed on a periapical radiograph without signs of inflammation (Fig. 6 and Y 2 M). A slight additional root resorption of the damaged tooth was observed in comparison with the post-treatment and 3 years 8months of follow-up panoramic radiographs (Figs. 8 and 13). Intraoral photographs showed stable occlusion with positive buccal overjet (Fig. 13).

## Discussion

To the best of our knowledge, immediate movement of a root-injured tooth towards the fractured root segment with pulp involvement has not yet been reported. Previously studies [14–17] described the orthodontic tooth movement of horizontal root-fractured incisors that initiated at least 2 years after injury in growing patients, while maintaining their vitality. Hwang et al. [7]. reported root perforation of the mandibular lateral incisor with pulp involvement after orthodontic miniscrew insertion. The perforated root was repaired surgically with mineral trioxide aggregate filling [7]. Lee et al. [10]. reported a maxillary premolar root fracture using an orthodontic miniscrew. The damaged tooth recovered its vitality 10 months after injury and was repaired spontaneously without complications [10]. Chang et al. [11]. reported that a mandibular first molar root fracture involving the pulp was repaired spontaneously with reparative tissue in growing patient. The separated root segment was resorbed while maintaining its vitality [11]. The authors reported no active movement of the damaged tooth [7, 10, 11].

According to studies of root repair after contact with orthodontic miniscrew, superficial root injury can be repaired by the deposition of cementum spontaneously after screw removal [6, 18, 19]. However, considering the severity of dental injury in this case, it was possible that the root fracture may give rise to irreversible reactions such as root resorption, ankylosis, or pulpal complications [6, 8, 20, 21]. Furthermore, the planned tooth movement was directed towards the fractured root segment. Hemisection of the fractured distal root or removal of the separated root segment was initially considered after endodontic treatment. Additional PDL damage resulting from surgical interventions may have also increased the risk of ankylosis during the healing process [13]. In the event of ankylosis, which would have led to the failure of the orthodontic movement, occurring after tooth injury or surgical intervention, the initial orthodontic treatment plan would have been modified to preserve the damaged tooth [22]. Consequently, the root-injured tooth movement was continued before the onset of possible ankylosis without an observation period or additional treatment.

Successful mandibular arch distalization was achieved without the ankylosis of the root-injured tooth (Figs. 7 and 10). Previous studies reported that the immediate application of orthodontic and occlusal forces stimulates the regeneration of the PDL in transplanted teeth [23, 24]. The continuation of the orthodontic treatment facilitated the healing of the damaged PDL, thereby enabling the root-injured tooth to move. Additionally, ankylosis is the process of a tooth being slowly replaced by bone and is related to the rate of bone turnover [25]. In this case, the patient was a 28-year-old who had a slower bone turnover rate than growing patient at the time of the root fracture [26].

The pulp vitality of the root-injured tooth was maintained for 6 years 2 months after injury. Although intermittent tooth hypersensitivity was observed during the active movement of the damaged tooth, endodontist recommended to monitor the condition of the tooth until the end of the orthodontic movement. Considering the miniscrew penetrated the middle of the distal root of the mandibular right first molar, the finding of the present case supports previous study that reported no evidence of pulp necrosis or inflammation in cases of severe root damage with pulp involvement in an animal study [27]. At the final follow-up visit, the patient was found to be asymptomatic.

Serial periapical radiographs and post-treatment CBCT revealed that the fractured root resorbed gradually during active tooth movement (Figs. 6 and 12). Inflammatory root resorption after tooth injury is caused by multinucleated cells that colonize the exposed dentin surface when the cementum is mechanically damaged [28]. The resorption process can be arrested spontaneously without additional stimulation [28]. Previous studies [25, 29] recommended observation periods of at least 1 year to prevent root resorption without active force application to the damaged tooth. As a result, root resorption of the root-injured tooth was inevitable because of the immediate application of orthodontic forces to prevent possible ankylosis.

A counterclockwise rotation of the mandible and the occlusal plane was observed in the superimposition of cephalometric tracings (Fig. 9). This was caused by the removal of incisal interference and the intrusive uprighting of the mandibular second molars as a result of the distal movement of the mandibular arch. The stability of the treatment results was confirmed in 3 years 8 months follow-up photographs and radiograph (Fig. 13). The root-injured tooth maintained its vitality with slight additional root resorption on both roots after retention. Periodic observation of the fractured tooth is required for the long-term prognosis of tooth vitality, additional root resorption and structural repair.

## Conclusions

The root-injured mandibular first molar maintained its vitality during remarkable orthodontic movement. This case suggests that immediate tooth movement of a fractured tooth can be a viable option to avoid ankylosis when the tooth injury occurred during the middle of orthodontic treatment. Careful monitoring and strategic management are required to ensure successful tooth movement in the event of unintentional root damage caused by orthodontic miniscrews. Moreover, it is essential that dental clinicians are aware of the anatomical limitations and take the necessary precautions when placing the orthodontic miniscrews. To prevent root injuries caused by miniscrew placement, it may be advisable to consider the use of extra-alveolar miniscrews or miniplates for mandibular arch distalization.

## Data Availability

All data generated or analyzed during this study are included in this published article.

## Abbreviations

CBCT: Cone-beam computed tomography
PDL: Periodontal ligament
